# Retraction: Rod Test in Percutaneous Nephrolithotomy: A Practical Guide to Selective Nephrostomy Placement

**DOI:** 10.7759/cureus.r261

**Published:** 2026-11-04

**Authors:** Chetan Sharma, Rahul Gupta, Haseeb Mohammad, Arti Mahajan, Kshitij Gupta

**Affiliations:** 1 Urology, Bee Enn General Hospital, Jammu, IND; 2 Urology, Government Medical College and Hospital, Jammu, Jammu, IND; 3 Urology, Government Medical College, Jammu, Jammu, IND; 4 Anesthesiology and Critical Care, Government Medical College, Jammu, Jammu, IND; 5 Medical Education, Acharya Shri Chander College of Medical Sciences and Hospital, Jammu, IND

This article has been retracted by the Editor-in-Chief due to concerns regarding the description of the study design and conduct. After publication, the authors informed the journal that a substantial part of the reported cohort was studied before the study period stated in the article and before the ethics approval cited in the article was obtained. The corresponding author agrees with the decision to retract.

